# 2-(Naphthalen-2-yl)azulene

**DOI:** 10.1107/S1600536810004897

**Published:** 2010-02-13

**Authors:** Jian Ye, Zi-Fa Shi, Chun-Lin Sun, Zhu-Guo Xu, Hao-Li Zhang

**Affiliations:** a State Key Laboratory of Applied Organic Chemistry and College of Chemistry and, Chemical Engineering, Lanzhou University, Lanzhou, Gansu, 730000, People’s Republic of China

## Abstract

In the title compound, C_20_H_14_, a naphthalene ring system is linked at the 2-position to the 2-C atom of the five-membered ring of an azulene unit. The compound crystallizes with two reasonably similar mol­ecules in the asymmetric unit. Neither mol­ecule is perfectly planar: the r.m.s. deviations from the best fit planes through all non-H atoms are 0.092 and 0.091 Å for the two mol­ecules. The dihedral angle between the mol­ecular planes is 49.60 (4)°. The naphthalene and azulene ring systems are inclined at dihedral angles of 6.54 (12) and 5.68 (12)° in the two mol­ecules. The crystal structure exhibits two sets of parallel layers, a typical edge-to-face herringbone packing arrangement. The structure is stabilized by an extensive series of weak C—H⋯π inter­actions.

## Related literature

For the structure and properties of azulene, see: Zhang & Petoud (2008 ▶); Dewar (1969 ▶); Wang *et al.* (1999 ▶). For applications of azulene derivatives, see: Ito *et al.* (2005 ▶); Lambert *et al.* (2003 ▶); Porsch *et al.* (1997 ▶); Schmitt *et al.* (1998 ▶). For the crystal structures of some organic semiconductors, see: Tan *et al.* (2009 ▶); Ando *et al.* (2005 ▶).
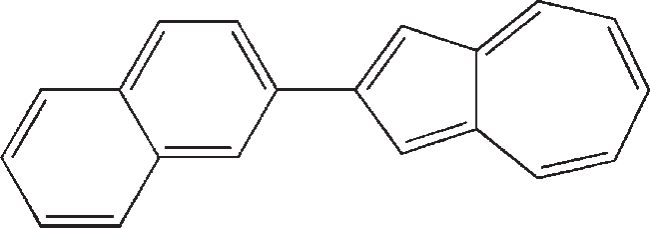


## Experimental

### 

#### Crystal data

C_20_H_14_
*M* *_r_* = 254.31
Triclinic, 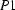

*a* = 6.0754 (8) Å
*b* = 7.6324 (11) Å
*c* = 14.5662 (19) Å
α = 97.997 (7)°
β = 97.179 (6)°
γ = 90.526 (7)°
*V* = 663.38 (16) Å^3^
*Z* = 2
Mo *K*α radiation
μ = 0.07 mm^−1^
*T* = 293 K
0.35 × 0.32 × 0.13 mm

#### Data collection

Bruker SMART CCD area-detector diffractometer
Absorption correction: multi-scan (*SADABS*; Bruker, 1997 ▶) *T*_min_ = 0.975, *T*_max_ = 0.991
3762 measured reflections
2573 independent reflections
2039 reflections with *I* > 2σ(*I*)
*R*_int_ = 0.021

#### Refinement

*R*[*F* ^2^ > 2σ(*F* ^2^)] = 0.050
*wR*(*F* ^2^) = 0.130
*S* = 1.04
2573 reflections
361 parameters
3 restraints
H-atom parameters constrained
Δρ_max_ = 0.16 e Å^−3^
Δρ_min_ = −0.16 e Å^−3^

### 

Data collection: *SMART* (Bruker 1997 ▶); cell refinement: *SAINT* (Bruker 1997 ▶); data reduction: *SAINT*; program(s) used to solve structure: *SHELXS97* (Sheldrick, 2008 ▶); program(s) used to refine structure: *SHELXL97* (Sheldrick, 2008 ▶); molecular graphics: *SHELXTL* (Sheldrick, 2008 ▶); software used to prepare material for publication: *SHELXTL*.

## Supplementary Material

Crystal structure: contains datablocks I, New_Global_Publ_Block. DOI: 10.1107/S1600536810004897/sj2726sup1.cif

Structure factors: contains datablocks I. DOI: 10.1107/S1600536810004897/sj2726Isup2.hkl

Additional supplementary materials:  crystallographic information; 3D view; checkCIF report

## Figures and Tables

**Table 1:** Hydrogen-bond geometry (Å, °) *Cg*1, *Cg*4, *Cg*5, *Cg*6, *Cg*7 and *Cg*8 are the centroids of the C1–C3/C8–C10, C13–C19, C1′–C2′/C7′–C10′, C2′–C7′, C11′–C13′/C19′,C20′ and C13′–C19′ rings, respectively.

*D*—H⋯*A*	*D*—H	H⋯*A*	*D*⋯*A*	*D*—H⋯*A*
C2—H2⋯*Cg*5^i^	0.93	2.84	3.488 (4)	128
C7—H7⋯*Cg*6^ii^	0.93	2.81	3.544 (4)	137
C8′—H8′⋯*Cg*1^iii^	0.93	2.82	3.456 (4)	126
C14—H14⋯*Cg*7^ii^	0.93	2.75	3.430 (4)	131
C14′—H14′⋯*Cg*4^iii^	0.93	2.77	3.485 (4)	135
C18—H18⋯*Cg*8^i^	0.93	2.73	3.446 (4)	134
C18′—H18′⋯*Cg*4	0.93	2.79	3.463 (4)	130

Symmetry codes: (i) 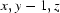; (ii) 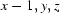; (iii) 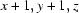.

**Fig. 1.**
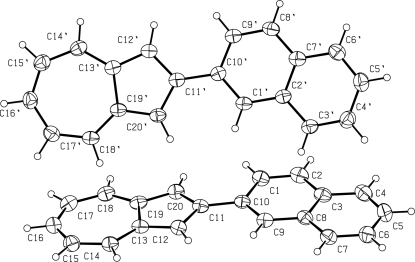
The asymmetric unit of (I) with displacement ellipsoids for the non-hydrogen atoms drawn at the 30% probability level.

**Fig. 2.**
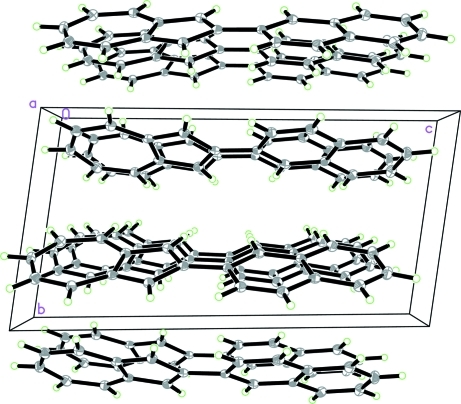
Crystal packing for (I) viewed down the a axis.

## supplementary crystallographic information

### Comment

Azulene, whose structure consists of a cyclopentadiene ring fused with a cycloheptatriene ring, is an isomer of naphthalene. However its photophysical properties differ significantly from those of naphthalene. Because of its particular electronic structure, azulene is a versatile organic fragment with both an electron-rich five-membered ring and an electron-deficient seven-membered ring (Zhang & Petoud, 2008). The calculations resulting from various aromaticity theories indicate that azulene possesses much lower aromatic delocalization energy (e.g., 4.2 kcal/mol) (Dewar, 1969, Wang *et al.*, 1999) compared to benzene, thiophene, and naphthalene. Due to this special character, azulene and its derivatives have attracted a growing interest in various areas of molecular materials, such as charge transfer complexes (Schmitt *et al.*, 1998), conducting polymers (Porsch *et al.*, 1997), liquid crystals (Ito *et al.*, 2005), as well as nonlinear optical (NLO) materials (Lambert *et al.*, 2003). This report on the crystal structure 2-(naphthalen-2-yl)azulene (I), is a preliminary report of our work on  the synthesis and analysis of azulene-containing molecular devices.

Compound I crystallizes with two discrete molecules in the asymmetric unit of the triclinic unit cell (space group P1). The torsion angle between azulene and naphthalene plane is 6.54 (12)° for one and 5.68 (12) ° for the other. The crystal packing of compound I can be classified as an edge-to-face herringbone-type geometry, and the tilt angle between two molecular planes is 49.60 (4)°. Such packing is similar to that of some organic semiconductors with oligomers (Tan *et al.*, 2009, Ando *et al.*, 2005).The crystal packing exhibits weak C—H···π interactions (Table 1). Cg1, Cg4, Cg5, Cg6, Cg7 and Cg8 are centroids of the C1—C2—C3—C8—C9—C10, C13—C19, C1'-C2'-C7'-C8'-C9'-C10', C2'—C7', C11'-C12'-C13'-C19'-C20' and C13'—C19' rings respectively.

### Experimental

To a mixture of azulen-2-yl trifluoromethanesulfonate (138 mg, 0.50 mmol) in THF (10 ml) were added 2-naphthalenylboronic acid (172 mg, 1.00 mmol), Pd(PPh_3_)_4_ (23 mg, 0.02 mmol) and Cs_2_CO_3_ saturated aqueous (1.0 ml). The mixture was refluxed overnight. The reaction was quenched with water, and extracted with ether (3 x 20 ml). Organic layers were washed with brine, dried (MgSO_4_), filtered, concentrated in vacuo and purified by flash chromatography (petroleum ether/CH_2_Cl_2_, 5:1) to provide the title compound as a dark-blue solid: mp 274–276 °C. Dark-blue crystalline blocks were obtained by slow evaporation of chloroform.

### Refinement

All H atoms were placed in geometrically idealized positions, with C—H = 0.93 Å, and constrained to ride on their respective parent atoms, with *U*_iso_(H) = 1.2*U*_eq_(C). In the absence of significant anomalous scattering effects, Friedel pairs were merged.

### Crystal data

**Table:**

C_20_H_14_	*Z* = 2
*M**_r_* = 254.31	*F*(000) = 268
Triclinic, *P*1	*D*_x_ = 1.273 Mg m^−^^3^
Hall symbol: P 1	Melting point = 547–549 K
*a* = 6.0754 (8) Å	Mo *K*α radiation, λ = 0.71073 Å
*b* = 7.6324 (11) Å	Cell parameters from 1303 reflections
*c* = 14.5662 (19) Å	θ = 2.9–24.7°
α = 97.997 (7)°	µ = 0.07 mm^−^^1^
β = 97.179 (6)°	*T* = 293 K
γ = 90.526 (7)°	Block, blue
*V* = 663.38 (16) Å^3^	0.35 × 0.32 × 0.13 mm

### Data collection

**Table:**

Bruker SMART CCD area-detector diffractometer	2573 independent reflections
Radiation source: fine-focus sealed tube	2039 reflections with *I* > 2σ(*I*)
graphite	*R*_int_ = 0.021
φ and ω scans	θ_max_ = 26.0°, θ_min_ = 1.4°
Absorption correction: multi-scan (*SADABS*; Bruker, 1997)	*h* = −7→7
*T*_min_ = 0.975, *T*_max_ = 0.991	*k* = −9→8
3762 measured reflections	*l* = −17→16

### Refinement

**Table:**

Refinement on *F*^2^	Primary atom site location: structure-invariant direct methods
Least-squares matrix: full	Secondary atom site location: difference Fourier map
*R*[*F*^2^ > 2σ(*F*^2^)] = 0.050	Hydrogen site location: inferred from neighbouring sites
*wR*(*F*^2^) = 0.130	H-atom parameters constrained
*S* = 1.04	*w* = 1/[σ^2^(*F*_o_^2^) + (0.0764*P*)^2^] where *P* = (*F*_o_^2^ + 2*F*_c_^2^)/3
2573 reflections	(Δ/σ)_max_ < 0.001
361 parameters	Δρ_max_ = 0.16 e Å^−^^3^
3 restraints	Δρ_min_ = −0.16 e Å^−^^3^

### Special details

**Table:**

Geometry. All esds (except the esd in the dihedral angle between two l.s. planes) are estimated using the full covariance matrix. The cell esds are taken into account individually in the estimation of esds in distances, angles and torsion angles; correlations between esds in cell parameters are only used when they are defined by crystal symmetry. An approximate (isotropic) treatment of cell esds is used for estimating esds involving l.s. planes.
Refinement. Refinement of *F*^2^ against ALL reflections. The weighted *R*-factor wR and goodness of fit *S* are based on *F*^2^, conventional *R*-factors *R* are based on *F*, with *F* set to zero for negative *F*^2^. The threshold expression of *F*^2^ > σ(*F*^2^) is used only for calculating *R*-factors(gt) etc. and is not relevant to the choice of reflections for refinement. *R*-factors based on *F*^2^ are statistically about twice as large as those based on *F*, and *R*- factors based on ALL data will be even larger.

### Fractional atomic coordinates and isotropic or equivalent isotropic displacement parameters (Å^2^)

**Table:**

	*x*	*y*	*z*	*U*_iso_*/*U*_eq_	
C1	0.3852 (6)	0.1216 (5)	0.5454 (3)	0.0531 (10)	
H1	0.4727	0.0839	0.4988	0.064*	
C2	0.4630 (7)	0.1084 (5)	0.6353 (3)	0.0576 (11)	
H2	0.6020	0.0617	0.6493	0.069*	
C3	0.3356 (7)	0.1650 (5)	0.7080 (3)	0.0526 (10)	
C4	0.4094 (7)	0.1490 (6)	0.8036 (3)	0.0630 (12)	
H4	0.5453	0.0992	0.8199	0.076*	
C5	0.2791 (9)	0.2075 (7)	0.8705 (3)	0.0711 (13)	
H5	0.3277	0.1972	0.9326	0.085*	
C6	0.0742 (8)	0.2825 (6)	0.8480 (3)	0.0653 (12)	
H6	−0.0116	0.3220	0.8949	0.078*	
C7	0.0002 (7)	0.2978 (5)	0.7577 (3)	0.0545 (10)	
H7	−0.1365	0.3482	0.7437	0.065*	
C8	0.1255 (6)	0.2393 (5)	0.6846 (3)	0.0471 (9)	
C9	0.0509 (6)	0.2508 (5)	0.5908 (3)	0.0480 (9)	
H9	−0.0859	0.3003	0.5759	0.058*	
C10	0.1729 (6)	0.1915 (4)	0.5200 (2)	0.0428 (9)	
C11	0.0910 (6)	0.1919 (5)	0.4208 (3)	0.0436 (9)	
C12	−0.1122 (6)	0.2601 (5)	0.3858 (3)	0.0479 (9)	
H12	−0.2134	0.3157	0.4223	0.057*	
C13	−0.1395 (6)	0.2324 (4)	0.2889 (3)	0.0443 (9)	
C14	−0.3185 (6)	0.2829 (5)	0.2301 (3)	0.0496 (9)	
H14	−0.4308	0.3389	0.2599	0.060*	
C15	−0.3528 (7)	0.2615 (6)	0.1338 (3)	0.0574 (11)	
H15	−0.4841	0.3057	0.1072	0.069*	
C16	−0.2149 (7)	0.1818 (6)	0.0712 (3)	0.0584 (11)	
H16	−0.2676	0.1809	0.0084	0.070*	
C17	−0.0131 (7)	0.1044 (6)	0.0883 (3)	0.0591 (11)	
H17	0.0498	0.0587	0.0354	0.071*	
C18	0.1091 (7)	0.0848 (5)	0.1718 (3)	0.0540 (10)	
H18	0.2418	0.0263	0.1675	0.065*	
C19	0.0620 (6)	0.1398 (5)	0.2616 (3)	0.0470 (9)	
C20	0.1931 (6)	0.1178 (5)	0.3448 (3)	0.0500 (9)	
H20	0.3286	0.0619	0.3488	0.060*	
C1'	0.5370 (6)	0.6436 (5)	0.5553 (3)	0.0456 (9)	
H1'	0.3975	0.5908	0.5352	0.055*	
C2'	0.6193 (6)	0.6635 (4)	0.6506 (3)	0.0440 (9)	
C3'	0.4909 (7)	0.6150 (5)	0.7189 (3)	0.0522 (10)	
H3'	0.3505	0.5633	0.6997	0.063*	
C4'	0.5691 (7)	0.6425 (6)	0.8116 (3)	0.0607 (11)	
H4'	0.4830	0.6079	0.8548	0.073*	
C5'	0.7779 (7)	0.7226 (6)	0.8422 (3)	0.0622 (11)	
H5'	0.8277	0.7446	0.9059	0.075*	
C6'	0.9095 (7)	0.7690 (5)	0.7797 (3)	0.0569 (10)	
H6'	1.0496	0.8198	0.8008	0.068*	
C7'	0.8332 (6)	0.7400 (5)	0.6821 (3)	0.0487 (9)	
C8'	0.9599 (6)	0.7907 (5)	0.6147 (3)	0.0536 (10)	
H8'	1.1029	0.8374	0.6333	0.064*	
C9'	0.8756 (6)	0.7720 (5)	0.5237 (3)	0.0486 (9)	
H9'	0.9626	0.8070	0.4809	0.058*	
C10'	0.6590 (6)	0.7009 (4)	0.4907 (2)	0.0420 (8)	
C11'	0.5689 (6)	0.6949 (4)	0.3917 (3)	0.0432 (9)	
C12'	0.6771 (6)	0.7610 (5)	0.3238 (3)	0.0467 (9)	
H12'	0.8171	0.8159	0.3342	0.056*	
C13'	0.5428 (6)	0.7320 (5)	0.2383 (3)	0.0456 (9)	
C14'	0.5962 (7)	0.7764 (5)	0.1547 (3)	0.0526 (10)	
H14'	0.7334	0.8335	0.1577	0.063*	
C15'	0.4729 (7)	0.7471 (6)	0.0676 (3)	0.0574 (11)	
H15'	0.5391	0.7871	0.0198	0.069*	
C16'	0.2669 (7)	0.6676 (6)	0.0410 (3)	0.0569 (10)	
H16'	0.2159	0.6595	−0.0224	0.068*	
C17'	0.1225 (7)	0.5972 (5)	0.0944 (3)	0.0549 (10)	
H17'	−0.0119	0.5511	0.0620	0.066*	
C18'	0.1523 (6)	0.5867 (5)	0.1894 (3)	0.0482 (9)	
H18'	0.0357	0.5345	0.2124	0.058*	
C19'	0.3336 (6)	0.6442 (4)	0.2542 (3)	0.0435 (8)	
C20'	0.3596 (6)	0.6260 (5)	0.3491 (3)	0.0459 (9)	
H20'	0.2543	0.5758	0.3795	0.055*	

### Atomic displacement parameters (Å^2^)

**Table:**

	*U*^11^	*U*^22^	*U*^33^	*U*^12^	*U*^13^	*U*^23^
C1	0.047 (2)	0.048 (2)	0.063 (3)	0.0042 (18)	0.0056 (19)	0.0059 (19)
C2	0.049 (2)	0.047 (2)	0.075 (3)	0.0051 (19)	0.003 (2)	0.007 (2)
C3	0.048 (2)	0.041 (2)	0.066 (3)	−0.0037 (18)	0.0014 (19)	0.0053 (18)
C4	0.055 (3)	0.061 (3)	0.070 (3)	−0.003 (2)	−0.007 (2)	0.011 (2)
C5	0.075 (3)	0.085 (3)	0.049 (3)	−0.009 (3)	−0.008 (2)	0.010 (2)
C6	0.062 (3)	0.077 (3)	0.054 (3)	−0.005 (2)	0.010 (2)	−0.001 (2)
C7	0.049 (2)	0.055 (2)	0.057 (3)	0.0003 (19)	0.0057 (18)	−0.0010 (19)
C8	0.049 (2)	0.036 (2)	0.053 (2)	−0.0037 (17)	0.0015 (17)	0.0011 (17)
C9	0.044 (2)	0.041 (2)	0.059 (2)	0.0024 (17)	0.0063 (17)	0.0077 (17)
C10	0.0410 (19)	0.0329 (19)	0.054 (2)	−0.0020 (16)	0.0063 (16)	0.0046 (16)
C11	0.038 (2)	0.0348 (19)	0.058 (2)	0.0015 (16)	0.0054 (16)	0.0067 (17)
C12	0.044 (2)	0.045 (2)	0.054 (3)	0.0057 (17)	0.0066 (17)	0.0051 (17)
C13	0.0359 (19)	0.0331 (18)	0.065 (3)	0.0057 (14)	0.0081 (16)	0.0081 (17)
C14	0.043 (2)	0.047 (2)	0.059 (3)	0.0061 (17)	0.0087 (17)	0.0080 (18)
C15	0.050 (2)	0.060 (3)	0.064 (3)	0.006 (2)	0.0025 (19)	0.016 (2)
C16	0.057 (3)	0.066 (3)	0.053 (2)	−0.003 (2)	0.0024 (19)	0.013 (2)
C17	0.059 (3)	0.059 (3)	0.063 (3)	0.001 (2)	0.018 (2)	0.012 (2)
C18	0.049 (2)	0.051 (2)	0.064 (3)	0.0026 (18)	0.0158 (19)	0.0096 (19)
C19	0.042 (2)	0.0363 (19)	0.065 (2)	0.0021 (15)	0.0154 (17)	0.0079 (17)
C20	0.046 (2)	0.046 (2)	0.059 (2)	0.0073 (18)	0.0093 (18)	0.0084 (18)
C1'	0.038 (2)	0.039 (2)	0.060 (2)	−0.0012 (16)	0.0065 (17)	0.0073 (17)
C2'	0.045 (2)	0.0330 (19)	0.055 (2)	0.0021 (16)	0.0100 (17)	0.0065 (16)
C3'	0.045 (2)	0.053 (2)	0.061 (3)	−0.0004 (18)	0.0114 (18)	0.0114 (19)
C4'	0.059 (3)	0.072 (3)	0.054 (3)	0.007 (2)	0.013 (2)	0.013 (2)
C5'	0.063 (3)	0.072 (3)	0.049 (2)	0.013 (2)	0.003 (2)	0.003 (2)
C6'	0.049 (2)	0.058 (3)	0.062 (3)	0.0041 (19)	0.0021 (19)	0.0034 (19)
C7'	0.043 (2)	0.041 (2)	0.062 (3)	0.0055 (17)	0.0073 (17)	0.0083 (17)
C8'	0.042 (2)	0.051 (2)	0.067 (3)	−0.0062 (18)	0.0022 (19)	0.0087 (19)
C9'	0.043 (2)	0.045 (2)	0.058 (2)	0.0000 (17)	0.0106 (17)	0.0067 (18)
C10'	0.045 (2)	0.0319 (18)	0.050 (2)	0.0036 (16)	0.0103 (16)	0.0042 (16)
C11'	0.042 (2)	0.0345 (19)	0.054 (2)	0.0047 (16)	0.0098 (16)	0.0065 (16)
C12'	0.041 (2)	0.044 (2)	0.056 (2)	−0.0016 (17)	0.0085 (17)	0.0050 (17)
C13'	0.044 (2)	0.0386 (19)	0.055 (2)	0.0039 (16)	0.0121 (17)	0.0044 (16)
C14'	0.053 (2)	0.050 (2)	0.057 (3)	0.0013 (18)	0.0178 (19)	0.0058 (18)
C15'	0.056 (3)	0.063 (3)	0.055 (3)	0.005 (2)	0.017 (2)	0.0069 (19)
C16'	0.059 (3)	0.068 (3)	0.042 (2)	0.008 (2)	0.0062 (18)	0.0022 (19)
C17'	0.047 (2)	0.059 (3)	0.056 (3)	0.0019 (19)	0.0024 (18)	0.0024 (19)
C18'	0.040 (2)	0.041 (2)	0.064 (3)	−0.0002 (16)	0.0087 (17)	0.0077 (17)
C19'	0.0365 (19)	0.0377 (19)	0.057 (2)	0.0035 (15)	0.0105 (16)	0.0057 (16)
C20'	0.038 (2)	0.043 (2)	0.058 (2)	0.0019 (16)	0.0082 (17)	0.0098 (17)

### Geometric parameters (Å, °)

**Table:**

C1—C2	1.353 (5)	C1'—C10'	1.382 (5)
C1—C10	1.426 (5)	C1'—C2'	1.402 (5)
C1—H1	0.9300	C1'—H1'	0.9300
C2—C3	1.411 (6)	C2'—C7'	1.415 (5)
C2—H2	0.9300	C2'—C3'	1.423 (5)
C3—C8	1.423 (5)	C3'—C4'	1.361 (6)
C3—C4	1.431 (6)	C3'—H3'	0.9300
C4—C5	1.362 (7)	C4'—C5'	1.397 (6)
C4—H4	0.9300	C4'—H4'	0.9300
C5—C6	1.395 (7)	C5'—C6'	1.365 (6)
C5—H5	0.9300	C5'—H5'	0.9300
C6—C7	1.357 (5)	C6'—C7'	1.425 (5)
C6—H6	0.9300	C6'—H6'	0.9300
C7—C8	1.410 (5)	C7'—C8'	1.412 (5)
C7—H7	0.9300	C8'—C9'	1.348 (5)
C8—C9	1.400 (5)	C8'—H8'	0.9300
C9—C10	1.373 (5)	C9'—C10'	1.418 (5)
C9—H9	0.9300	C9'—H9'	0.9300
C10—C11	1.469 (5)	C10'—C11'	1.471 (5)
C11—C20	1.391 (5)	C11'—C12'	1.401 (5)
C11—C12	1.408 (5)	C11'—C20'	1.407 (5)
C12—C13	1.386 (5)	C12'—C13'	1.390 (5)
C12—H12	0.9300	C12'—H12'	0.9300
C13—C14	1.389 (5)	C13'—C14'	1.384 (5)
C13—C19	1.488 (5)	C13'—C19'	1.490 (5)
C14—C15	1.379 (5)	C14'—C15'	1.379 (5)
C14—H14	0.9300	C14'—H14'	0.9300
C15—C16	1.395 (6)	C15'—C16'	1.372 (6)
C15—H15	0.9300	C15'—H15'	0.9300
C16—C17	1.376 (6)	C16'—C17'	1.394 (6)
C16—H16	0.9300	C16'—H16'	0.9300
C17—C18	1.371 (6)	C17'—C18'	1.386 (5)
C17—H17	0.9300	C17'—H17'	0.9300
C18—C19	1.383 (5)	C18'—C19'	1.383 (5)
C18—H18	0.9300	C18'—H18'	0.9300
C19—C20	1.396 (5)	C19'—C20'	1.398 (5)
C20—H20	0.9300	C20'—H20'	0.9300
			
C2—C1—C10	122.0 (4)	C10'—C1'—C2'	121.3 (3)
C2—C1—H1	119.0	C10'—C1'—H1'	119.3
C10—C1—H1	119.0	C2'—C1'—H1'	119.3
C1—C2—C3	120.7 (4)	C1'—C2'—C7'	120.0 (3)
C1—C2—H2	119.7	C1'—C2'—C3'	122.1 (3)
C3—C2—H2	119.7	C7'—C2'—C3'	117.8 (4)
C2—C3—C8	118.4 (4)	C4'—C3'—C2'	121.4 (4)
C2—C3—C4	122.4 (4)	C4'—C3'—H3'	119.3
C8—C3—C4	119.2 (4)	C2'—C3'—H3'	119.3
C5—C4—C3	119.4 (4)	C3'—C4'—C5'	120.3 (4)
C5—C4—H4	120.3	C3'—C4'—H4'	119.8
C3—C4—H4	120.3	C5'—C4'—H4'	119.8
C4—C5—C6	121.5 (4)	C6'—C5'—C4'	120.6 (4)
C4—C5—H5	119.2	C6'—C5'—H5'	119.7
C6—C5—H5	119.2	C4'—C5'—H5'	119.7
C7—C6—C5	120.1 (4)	C5'—C6'—C7'	120.3 (4)
C7—C6—H6	120.0	C5'—C6'—H6'	119.9
C5—C6—H6	120.0	C7'—C6'—H6'	119.9
C6—C7—C8	121.6 (4)	C8'—C7'—C2'	118.0 (4)
C6—C7—H7	119.2	C8'—C7'—C6'	122.5 (4)
C8—C7—H7	119.2	C2'—C7'—C6'	119.4 (4)
C9—C8—C7	122.7 (3)	C9'—C8'—C7'	120.7 (4)
C9—C8—C3	119.2 (4)	C9'—C8'—H8'	119.7
C7—C8—C3	118.1 (4)	C7'—C8'—H8'	119.7
C10—C9—C8	122.3 (3)	C8'—C9'—C10'	122.5 (3)
C10—C9—H9	118.9	C8'—C9'—H9'	118.8
C8—C9—H9	118.9	C10'—C9'—H9'	118.8
C9—C10—C1	117.5 (3)	C1'—C10'—C9'	117.4 (3)
C9—C10—C11	122.9 (3)	C1'—C10'—C11'	122.2 (3)
C1—C10—C11	119.5 (3)	C9'—C10'—C11'	120.3 (3)
C20—C11—C12	107.7 (3)	C12'—C11'—C20'	108.1 (3)
C20—C11—C10	126.5 (3)	C12'—C11'—C10'	125.3 (3)
C12—C11—C10	125.7 (3)	C20'—C11'—C10'	126.5 (3)
C13—C12—C11	110.1 (3)	C13'—C12'—C11'	109.4 (3)
C13—C12—H12	124.9	C13'—C12'—H12'	125.3
C11—C12—H12	124.9	C11'—C12'—H12'	125.3
C12—C13—C14	126.5 (3)	C14'—C13'—C12'	125.9 (4)
C12—C13—C19	105.9 (3)	C14'—C13'—C19'	127.1 (4)
C14—C13—C19	127.6 (4)	C12'—C13'—C19'	107.0 (3)
C15—C14—C13	129.0 (4)	C15'—C14'—C13'	128.5 (4)
C15—C14—H14	115.5	C15'—C14'—H14'	115.7
C13—C14—H14	115.5	C13'—C14'—H14'	115.7
C14—C15—C16	128.1 (4)	C16'—C15'—C14'	129.5 (4)
C14—C15—H15	116.0	C16'—C15'—H15'	115.3
C16—C15—H15	116.0	C14'—C15'—H15'	115.3
C17—C16—C15	129.9 (4)	C15'—C16'—C17'	130.0 (4)
C17—C16—H16	115.1	C15'—C16'—H16'	115.0
C15—C16—H16	115.1	C17'—C16'—H16'	115.0
C18—C17—C16	129.6 (4)	C18'—C17'—C16'	128.7 (4)
C18—C17—H17	115.2	C18'—C17'—H17'	115.7
C16—C17—H17	115.2	C16'—C17'—H17'	115.7
C17—C18—C19	128.9 (4)	C19'—C18'—C17'	128.2 (4)
C17—C18—H18	115.6	C19'—C18'—H18'	115.9
C19—C18—H18	115.6	C17'—C18'—H18'	115.9
C18—C19—C20	126.8 (4)	C18'—C19'—C20'	126.2 (3)
C18—C19—C13	126.9 (4)	C18'—C19'—C13'	128.1 (3)
C20—C19—C13	106.3 (3)	C20'—C19'—C13'	105.6 (3)
C11—C20—C19	109.9 (3)	C19'—C20'—C11'	109.8 (3)
C11—C20—H20	125.0	C19'—C20'—H20'	125.1
C19—C20—H20	125.0	C11'—C20'—H20'	125.1
			
C10—C1—C2—C3	−0.3 (6)	C10'—C1'—C2'—C7'	−1.4 (5)
C1—C2—C3—C8	−1.2 (6)	C10'—C1'—C2'—C3'	176.2 (3)
C1—C2—C3—C4	178.3 (4)	C1'—C2'—C3'—C4'	−176.9 (4)
C2—C3—C4—C5	179.5 (4)	C7'—C2'—C3'—C4'	0.7 (5)
C8—C3—C4—C5	−1.0 (6)	C2'—C3'—C4'—C5'	1.1 (6)
C3—C4—C5—C6	0.2 (7)	C3'—C4'—C5'—C6'	−2.2 (7)
C4—C5—C6—C7	0.3 (8)	C4'—C5'—C6'—C7'	1.5 (7)
C5—C6—C7—C8	0.1 (7)	C1'—C2'—C7'—C8'	−1.4 (5)
C6—C7—C8—C9	178.9 (4)	C3'—C2'—C7'—C8'	−179.1 (3)
C6—C7—C8—C3	−0.9 (6)	C1'—C2'—C7'—C6'	176.3 (3)
C2—C3—C8—C9	1.1 (5)	C3'—C2'—C7'—C6'	−1.4 (5)
C4—C3—C8—C9	−178.4 (4)	C5'—C6'—C7'—C8'	177.9 (4)
C2—C3—C8—C7	−179.1 (4)	C5'—C6'—C7'—C2'	0.3 (6)
C4—C3—C8—C7	1.3 (5)	C2'—C7'—C8'—C9'	2.3 (5)
C7—C8—C9—C10	−179.2 (4)	C6'—C7'—C8'—C9'	−175.4 (3)
C3—C8—C9—C10	0.5 (5)	C7'—C8'—C9'—C10'	−0.3 (6)
C8—C9—C10—C1	−2.0 (5)	C2'—C1'—C10'—C9'	3.3 (5)
C8—C9—C10—C11	175.9 (3)	C2'—C1'—C10'—C11'	−174.8 (3)
C2—C1—C10—C9	1.9 (5)	C8'—C9'—C10'—C1'	−2.5 (5)
C2—C1—C10—C11	−176.0 (3)	C8'—C9'—C10'—C11'	175.7 (3)
C9—C10—C11—C20	−172.8 (4)	C1'—C10'—C11'—C12'	175.6 (4)
C1—C10—C11—C20	5.0 (5)	C9'—C10'—C11'—C12'	−2.4 (5)
C9—C10—C11—C12	3.7 (5)	C1'—C10'—C11'—C20'	−3.0 (5)
C1—C10—C11—C12	−178.5 (4)	C9'—C10'—C11'—C20'	178.9 (4)
C20—C11—C12—C13	−0.7 (4)	C20'—C11'—C12'—C13'	−1.3 (4)
C10—C11—C12—C13	−177.7 (3)	C10'—C11'—C12'—C13'	179.8 (3)
C11—C12—C13—C14	−179.4 (3)	C11'—C12'—C13'—C14'	−178.7 (4)
C11—C12—C13—C19	0.0 (4)	C11'—C12'—C13'—C19'	1.1 (4)
C12—C13—C14—C15	178.8 (4)	C12'—C13'—C14'—C15'	178.2 (4)
C19—C13—C14—C15	−0.5 (6)	C19'—C13'—C14'—C15'	−1.5 (7)
C13—C14—C15—C16	0.5 (7)	C13'—C14'—C15'—C16'	0.2 (8)
C14—C15—C16—C17	0.3 (8)	C14'—C15'—C16'—C17'	1.4 (8)
C15—C16—C17—C18	−0.3 (8)	C15'—C16'—C17'—C18'	−1.2 (8)
C16—C17—C18—C19	−0.8 (7)	C16'—C17'—C18'—C19'	0.1 (7)
C17—C18—C19—C20	−179.4 (4)	C17'—C18'—C19'—C20'	−177.9 (4)
C17—C18—C19—C13	1.3 (7)	C17'—C18'—C19'—C13'	0.0 (6)
C12—C13—C19—C18	180.0 (4)	C14'—C13'—C19'—C18'	1.1 (6)
C14—C13—C19—C18	−0.6 (6)	C12'—C13'—C19'—C18'	−178.7 (3)
C12—C13—C19—C20	0.6 (4)	C14'—C13'—C19'—C20'	179.3 (4)
C14—C13—C19—C20	−179.9 (3)	C12'—C13'—C19'—C20'	−0.4 (4)
C12—C11—C20—C19	1.1 (4)	C18'—C19'—C20'—C11'	177.9 (3)
C10—C11—C20—C19	178.1 (3)	C13'—C19'—C20'—C11'	−0.4 (4)
C18—C19—C20—C11	179.6 (4)	C12'—C11'—C20'—C19'	1.0 (4)
C13—C19—C20—C11	−1.1 (4)	C10'—C11'—C20'—C19'	179.9 (3)

### Hydrogen-bond geometry (Å, °)

**Table:**

Cg1, Cg4, Cg5, Cg6, Cg7 and Cg8 are the centroids of the C1–C3,C8–C10, C13–C19, C1'-C2',C7'–C10', C2'–C7', C11'–C13',C19',C20' and C13'–C19' rings, respectively.

**Table:**

*D*—H···*A*	*D*—H	H···*A*	*D*···*A*	*D*—H···*A*
C2—H2···Cg5^i^	0.93	2.84	3.488 (4)	128
C7—H7···Cg6^ii^	0.93	2.81	3.544 (4)	137
C8'—H8'···Cg1^iii^	0.93	2.82	3.456 (4)	126
C14—H14···Cg7^ii^	0.93	2.75	3.430 (4)	131
C14'—H14'···Cg4^iii^	0.93	2.77	3.485 (4)	135
C18—H18···Cg8^i^	0.93	2.73	3.446 (4)	134
C18'—H18'···Cg4	0.93	2.79	3.463 (4)	130

Symmetry codes: (i) *x*, *y*−1, *z*; (ii) *x*−1, *y*, *z*; (iii) *x*+1, *y*+1, *z*.
